# Using the ICH estimand framework to improve the interpretation of treatment effects in internet interventions

**DOI:** 10.1038/s41746-025-01936-0

**Published:** 2025-08-20

**Authors:** Manuel Heinrich, Pavle Zagorscak, Johannes Bohn, Christine Knaevelsrud, Lars Schulze

**Affiliations:** 1 Division of Clinical-Psychological Intervention, Department of Education and Psychology, Freie Universität Berlin, Berlin, Germany; 2 German Center for Mental Health (DZPG), partner site Berlin-Potsdam, Berlin-Potsdam, Germany; 3 Division of Clinical Psychology and Psychotherapy, Department of Education and Psychology, Freie Universität Berlin, Berlin, Germany

**Keywords:** Outcomes research, Clinical trial design

## Abstract

The ICH E9(R1) *Addendum on Estimands and Sensitivity Analysis* provides a framework for defining the treatment effect a trial intends to estimate—the estimand. The addendum is widely adopted in pharmaceutical research. However, it remains underutilized in trials investigating internet-based interventions (IBIs). This manuscript introduces the addendum to IBI researchers. It concludes that estimands are essential to improve the interpretability, relevance, and validity of effect estimates derived in IBI trials.

## Introduction

To ensure that randomized controlled trials of (non-) digital (mental) health interventions yield informative results, researchers need to define the intended substantive meaning of the treatment effect they intend to estimate: the *estimand*^1–4^. An estimand forms the basis for designing trials that allow collecting data needed to answer the clinical question. An estimator (i.e., a statistical procedure) that is aligned with the estimand is used to summarize the data and derive an estimate that accurately quantifies the estimand^1,3,4^.

The ICH E9(R1) *Addendum on Estimands and Sensitivity Analyses*, developed by regulatory authorities in the pharmaceutical industry, was published to facilitate the process of defining estimands^1^. Essentially, it recommends three steps^1,3^:

1. Define the estimand attributes: population, endpoint, treatment, and statistical summary measure^1^.
2. Identify possible intercurrent events (ICEs): events that occur after treatment initiation and affect either the interpretation or availability of measurements relevant to answering the clinical question (e.g., treatment discontinuation)^1^.
3. Choose strategies for dealing with ICEs that ensure that the estimate remains aligned with the estimand^1^.

Pharmacology researchers have adopted the addendum; numerous publications explained, refined, and illustrated its application^3–19^. However, the addendum is underutilized in Internet-based interventions (IBIs: interventions that provide individuals access to evidence-based psychotherapeutic techniques in a digital format) research, despite its potential to (1) enhance the interpretability of IBI studies^4,20^ and (2) its recognition in the European Medicines Agency (EMA) guidelines on depression trials^21^ and the updated CONSORT guidelines^22^. This manuscript provides an overview of the estimand framework for IBI researchers, particularly clinical psychologists, psychiatrists, and non-statistical professionals who play a key role in trial design and implementation. We outline key assumptions and provide resources for statistical details.

## Motivating example

Two trials evaluate the same IBI for depression. They use the same inclusion criteria, recruit from routine care, employ a waitlist control, and use self-reported depressive symptoms 8 weeks after randomization as the endpoint.

*Trial A* assessed depressive symptoms weekly, but only while patients used the IBI. Thus, the endpoint is missing for individuals who discontinued treatment. Trial A replaces missing endpoints using multiple imputations with depression scores collected while individuals used the IBI as auxiliary information. *Trial B* collects the endpoint from *all* patients, even if they discontinued the IBI. Thus, Trial B has no missing values.

Since neither study defines what treatment effect they intend to estimate or describes the assumptions underlying their approaches, the reader must infer the meaning of the estimated treatment effect from how the data were collected and analyzed^4^. It becomes evident that the two trials answer *different* clinical questions.

Since the imputation model in Trial A only knows how the symptoms develop while individuals are on treatment, it imputes the missing values of discontinuers as if they continued the treatment^23,24^. Trial A estimates a treatment effect for a *hypothetical scenario* in which one has found a “magical” measure to get all non-adherers to adhere^24,25^. However, the assumption that individuals who discontinued IBI have similar symptom trajectories to those who completed it, especially if discontinuation is related to the intervention, worsening of symptoms, or other characteristics that distinguish discontinuers from those who adhere, is unrealistic. Trial A appears to be valid. However, not collecting endpoints from discontinuers can be a trial-related flaw that may lead to overestimated treatment effects that do not generalize to clinically relevant contexts.

Trial B collects the endpoint from all patients, regardless of adherence. It targets the symptom change in a *real-world scenario* in which treatment discontinuation occurs.

## The estimand, its attributes, and intercurrent events

The estimand is the central concept of the ICH E9(R1) addendum. An *estimand* is a systematic description of the effect researchers want to quantify^1,3^. The addendum recommends defining the estimand along five attributes^1,3^.

- *Treatment:* A complete description of the treatment regimen for all study arms is required, including all digital (e.g., number of modules, intended dosage) and non-digital components (e.g., whether concurrent antidepressant use is permitted, emergency procedures). It is incorrect to equate the treatment with the IBI; the IBI is typically just the major ingredient of a comprehensive treatment regimen.
- *Population:* The eligibility criteria define the target population^1^. Baseline characteristics inform how well the sample represents it. The addendum highlights the possibility of focusing on principal strata (see section on *principal strata*)^1,3^.
- *Endpoint:* The endpoint is the variable collected to quantify the treatment effect, including the assessment time and modality^1^. Endpoints may be questionnaire scores, diagnostic classifications, or composite variables^1^.
- *Population-level summary:* The population-level summary is a statistical measure calculated to quantify the treatment effect^1,3^. IBI trials are typically interested in differences in mean symptom scores or responder rates.

Table 1 summarizes some considerations for defining these attributes in IBI trials. These aspects are not IBI-specific, but important due to the nature of IBIs.

**Table 1:** Aspects related to the four main attributes of the estimand in trials investigating IBIs

**Treatment**
►*Describe all components of the IBI*. IBIs are treatment packages that differ in several dimensions, such as (a) the theoretical foundation, (b) the number of modules, (c) the psychotherapeutic techniques used, (d) the sequence and/or the timing of treatment modules, (e) the rules for how clients access new content, (f) the amount and type of guidance, or (g) the type of personalization. Trial protocols should describe all components. This description should also include aspects of technical implementation, such as (i) the type of platform, (ii) the form of the application (mobile vs. web-based), and (iii) the nudging mechanisms used (e.g., gamification, reminders). ►*Define what “being treated” means*. In principle, various meaningful definitions exist, such as (a) gaining access to the treatment material or (b) completing all or a certain proportion of the modules. However, IBI participants often choose the dose and intensity of the IBI treatment for themselves. This IBI characteristic makes some estimands more plausible (e.g., the treatment effect of individuals gaining access to an IBI with a non-standardized and self-selected dose; *treatment policy strategy*) than others (e.g., the effect that would have been observed with perfect adherence). Moreover, protocol deviations are difficult to define and assess. This also concerns treatment discontinuation. For example, in an IBI trial where participants enter modules sequentially, a treatment could be considered discontinued if individuals stop logging in to the modules. However, in other IBIs, individuals decide for themselves how many and which modules they use; defining discontinuation and handling it appropriately can become much more challenging. Consequently, many IBI trials likely focus on the effect of making an IBI accessible. ►*Define if the treatment regimen under investigation accepts treatments in parallel*. IBIs are low-threshold interventions that are not regularly accompanied by visits to study centers. Therefore, there is little control over what individuals do in parallel. In the eligibility criteria, IBI often limits the parallel treatments with which participants are allowed to enter the trial. However, the use of parallel treatments initiated *after* randomization is usually not restricted. Consequently, it should be explicitly stated which parallel treatments are allowed. In most instances, especially in clinical samples, all parallel treatments are allowed and a *treatment policy strategy* (e.g., collect data even if individuals use treatments in parallel) will be employed. However, this should be stated explicitly, and the frequency and intensity of parallel treatments should be assessed and reported.
**Population**
►*Excluded post-hoc changes the population attribute*. IBI trials may exclude randomized patients post hoc (*modified* intention-to-treat analysis^53^). In IBI trials, this may concern individuals who have at least started working with the program or completed at least two modules. However, such exclusions change the population attribute, may introduce bias^54^, and must be justified. Consider the exclusion of individuals who commit suicide immediately after randomization. This exclusion is only justifiable if the researcher is absolutely certain that the observed adverse event has nothing to do with the outcome of the randomization (e.g., if individuals commit suicide because they were assigned to a treatment arm that they consider useless); if applied inappropriately, one throws away evidence against the specific treatment regimen that caused the adverse event. This also applies to per-protocol analyses that define strata based on observed completion status (see Table 3).
**Endpoint**
►*Consider the time-point of assessment as an endpoint attribute*. Describe when the endpoint will be assessed. If possible, define a time frame that is considered acceptable and how observations collected outside this time frame are handled. ►*Discuss whether linking measurement to progress in the IBI is sensible*. IBI trials may align assessments with specific events, such as the start of a new module or completion of all modules. However, this creates a correlation between adherence and endpoint availability, which raises methodological challenges. First, assessment times can vary widely when participants complete the program at their own pace, making it difficult to control for time effects. Second, if the assessment of the outcome is linked to the completion of the intervention, participants control when the endpoint is assessed; for example, participants may complete the endpoint assessment when they feel ready rather than at the intended point in time^55^. If the missing assessments at the time of interest depend on the endpoint itself, the estimate could be biased^55,56^. ►*Assessing endpoints outside the IBI could be useful*. While conventional clinical trials often involve on-site assessments conducted in study centers, this is less common in IBI trials. IBIs may assess the endpoint within the application. However, this can result in a correlation between endpoint availability and adherence. Especially if no information is available about how endpoints developed among individuals who discontinued the IBI, problems can arise. Researchers must rely on a hypothetical strategy that imposes assumptions about the post-ICE symptom development. Therefore, alternative assessment modalities (e.g., telephone interviews) for the primary endpoint should be considered. This method allows for standardization of the assessment time-point for all participants, reduces reliance on IBI participation patterns, and could increase the sense of commitment to the evaluations. ►*Apply the same principles to endpoints different from symptom change*. In some studies, other variables, such as treatment discontinuation, could be the endpoints. To derive meaningful treatment effects, the same principles discussed for defining appropriate estimands should be applied.
**Statistical summary measures**
► *Select an effect size measure that is aligned with the estimand*. If a trial is concerned with the mean difference, the statistical summary measure should reflect this mean difference. Therefore, specifying effect sizes that reflect the proportion of variance explained is not informative (e.g., R^2^). ►*State your standardizer*. Many IBI studies report between-group effects in standardized mean differences. If standardization is required, the standardizer should be specified. For a given mean difference, different standardizers lead to differences in the size of the effect sizes^57^ and, more importantly, represent different estimands^58^. Standardizing a mean difference by the standard deviation of an untreated reference population has a different meaning than standardizing by the standard deviation of endpoint among all randomized individuals at the endpoint assessment^59^. It may be useful to report effects in units of the scale, as raw, unstandardized scale values do not depend on the standardizer and are often informative to clinicians.

The first four attributes help to identify relevant *intercurrent events* (ICEs), defined as “events occurring after treatment initiation that affect either [1] the interpretation or [2] the existence of the measurements associated with the clinical question of interest”^1^. Researchers should anticipate ICEs and discuss how they relate to the four attributes^1,3^. ICEs that result in measurements that misalign with one of the attributes are relevant.

Consider a trial that intends to estimate the effect of a scenario in which all individuals use IBI as intended versus no treatment (*treatment*) among adults with depression (*population*) in terms of mean differences (*population level summary*) in PHQ-9 scores 8 weeks after randomization (*endpoint*). Some patients are expected to discontinue the treatment (*ICE*). Treatment discontinuation fulfills both criteria of an ICE. First, it affects the interpretation of measurements collected after discontinuation. These measurements reflect the effects of the IBI and everything that happened after discontinuation. Second, measurements necessary to answer the clinical question do not exist for individuals who discontinued the treatment because they did not complete it. To keep the estimate aligned with the estimand, measurements affected by the ICE must be handled properly. This leads to the fifth attribute: *the strategies for handling ICE*.

- *Handling ICEs*: Researchers should handle ICEs in a manner that keeps the estimate aligned with the estimand^1,3^. The addendum outlines five strategies, which can be combined: (1) treatment policy strategy, (2) hypothetical strategy, (3) while-on-treatment strategy, (4) composite strategy, and (5) principal strata strategy^1,3^.

The addendum highlights the difference between data affected by an ICE and missing data^1,3^. Missing data refers to information that was available but has not been collected. In our example, missing data results when individuals who complete the treatment are not assessed. This is different from data affected by an ICE. In our discontinuation example, the data cannot be collected because the individuals have not completed the IBI.

## Strategies to treat intercurrent events

The addendum introduces a fundamental principle: *whenever an ICE occurs, the post-ICE data, whether observed or missing, must be handled in a way that aligns with the estimand*^1^. This section provides a conceptual overview of the five strategies for handling ICEs suggested in the addendum. There is no one-size-fits-all approach. Each strategy entails trade-offs. The appropriate (combined) strategy depends on the clinical question^3,8^. How to handle ICEs should be decided in a comprehensive discussion among all stakeholders^1^. All strategies and statistical methods employed to implement the strategies rely on assumptions. These assumptions should be stated^4,7^. Sensitivity analyses are necessary to assess the robustness of conclusions against violations of these assumptions^1,3^.

We focus on treatment discontinuation (ICE) in a simplified treatment regimen that comprises an IBI as the main treatment ingredient. Participants work with eight modules (one per week) in sequential order. We consider the treatment “discontinued” if the person no longer logs in in spite of not yet having completed all available treatment modules. We use this simplified example to illustrate how different strategies alter the interpretation of the treatment effects. Table 2 illustrates the application of the strategies to other ICEs. Table 3 relates the strategies to concepts widely used in IBI research: (a) the intention-to-treat principle, (b) per-protocol analysis, and (c) efficacy vs. effectiveness trials.

**Table 2:** Further examples of applying the strategies to different intercurrent events

**Example 1:** A trial aims to estimate the effect of providing individuals access to an IBI in comparison to no treatment in a scenario where no antidepressants are available. Some individuals in the IBI arm will start antidepressants in parallel (=ICE), initiated outside the trial. For these individuals, an antidepressant-free assessment will not be available. A *hypothetical strategy* could be employed to model a scenario in which all participants have completed the IBI without taking antidepressants. Researchers must assume that data from participants who did not start antidepressants is sufficient to model symptom development under the hypothetical scenario. However, individuals who start antidepressants may differ from those who do not initiate antidepressants; covariates to adjust for this may not be observed. If the MAR assumption does not hold, the estimates will be biased. If starting antidepressants in parallel is common clinical practice, the estimate lacks clinical relevance.
**Example 2:** A study aims to evaluate the effect of a treatment regimen that comprises an IBI targeting depression in patients undergoing cancer treatment. Some participants may discontinue the intervention (=ICE) due to a deteriorating somatic condition unrelated to the treatment. First, suppose the depressive severity at a fixed time point is of interest (e.g., 3 months after randomization). One may model the missing assessments by focusing on the hypothetical scenario in which individuals could have continued the treatment (*hypothetical strategy*). It is necessary to assume that the available information collected among individuals who completed the IBI is sufficient to recover the symptom course under this hypothetical scenario. The estimate quantifies a scenario in which the somatic condition did not deteriorate. Second, researchers could also try to follow up with individuals despite their deteriorating somatic symptoms and consider deteriorations followed by discontinuation a natural characteristic of the target population (*treatment policy strategy*). The latter approach assumes that individuals can benefit from the psychotherapeutic techniques they learn when working with IBI, even after they discontinue the treatment due to the worsening somatic condition. However, this becomes problematic when the worsening somatic condition leads to death. *Lastly*, one may use a *while-on-treatment* approach, focusing on the symptom while individuals are using the treatment.
**Example 3:** In a trial investigating an IBI for adults with depression, some individuals will report elevated levels of suicidality, necessitating acute inpatient treatment. First, one could treat hospitalization as part of the treatment regimen and attempt to follow up with all participants (*treatment policy strategy*). This seems to provide a clinically relevant estimate. However, the resulting estimate is a blend of the IBI and hospitalization. If the hospitalization rates differ between treatment arms, problems can arise in interpreting the effect of the treatment. Alternatively, hospitalization can be defined as a “treatment failure” and used as a component of a composite endpoint (*composite strategy*). However, the composite strategy assumes that hospitalization has the same clinical relevance as other “treatment failure” indicators merged in the composite.
**Example 4:** A study compares an IBI to face-to-face psychotherapy (f2f-PT). The depression severity 12 months after randomization is the endpoint. The stakeholder expected that some participants in the IBI group may start psychotherapy between the end of the IBI and the 12-month assessment (ICE). *First*, researchers could decide to assess all individuals regardless of whether they have started f2f-PT after the IBI (*treatment policy strategy*). This approach would effectively compare IBI with the possibility of face-to-face psychotherapy if needed, to standard f2f-PT. *Second*, researchers could discard the assessments of individuals who began f2f-PT after IBI and attempt to model a scenario in which these individuals did not access f2f-PT (*hypothetical strategy*). Applying the hypothetical strategy can be challenging when no follow-up information is available for individuals without access to f2f-PT. Then, it would be necessary to rely solely on assumptions about symptom development in this hypothetical scenario. If, however, such information is available, one could assume that symptom development is similar to that of those who did not access f2f-PT, although their follow-up measurements suggest that they may have benefited from participating in f2f-PT. The same restriction as in Example 1 applies: if the MAR condition is violated, the estimates will be biased. *Third*, individuals who entered f2f-PT after IBI could be considered “treatment failures” in a composite measure (*composite strategy*). Again, the merged indicators of treatment failure must have the same clinical relevance. *Last*ly, one could focus on the effect among patients who would use the assigned treatment as intended (i.e., either f2f-PT or IBI without subsequent f2f-PT), regardless of their assigned treatment (principal stratum). However, the researchers need to assume that sufficient data is available to recover the latent strata with sufficient accuracy.

**Table 3:** Relation to other important concepts in IBI research

**Intention-to-treat (ITT):** Many IBI trials intend to adhere to the ITT principle. The understanding of an ITT analysis is typically centered around including all randomized patients in the analysis and (b) maintaining random group assignment^60^. However, these characteristics leave ambiguity about the estimated effect without considering (c) the handling of ICEs^60^. Recall our motivating example of treatment discontinuation. Trial A, did not assess individuals after treatment discontinuation, and missing values are imputed targeting a hypothetical strategy. Trial B collects the outcome of all randomized individuals. Both trials adhere to principles (a) and (b). However, they target different estimands, as they handle the ICE differently. This ambiguity can be avoided if the following information is provided: (1) Is the primary endpoint collected from all randomized individuals—even if an ICE occurred? (2) Are measurements affected by an ICE discarded or retained in the analysis? (3) How does the statistical approach used to address missing endpoints account for the ICE?
**Per-protocol analysis (PPA):** The PPA typically compares patients who have completed the treatment according to specific adherence criteria (e.g., at least 75% of the modules completed, where ‘completed’ in IBI trials normally means that individuals entered the modules and worked with them at a self-selected dose). However, the PPA is likely biased because randomization no longer holds^17,39,54,61^. A hypothetical (i.e., impute values of non-adherent individuals as *if they were adherent;* target population: all randomized individuals) or the principal strata strategy (target population: individuals *who would complete the treatment*, irrespective of which treatment they are assigned) can address the question of PPA^17,39,61^. Both strategies make strong assumptions but may avoid the biases ingrained in PPA^17^. It is an ongoing discussion about which approach is the best^e.g.,^ ^10,61,62^.
**Efficacy and effectiveness:** In psychotherapeutic trials, “efficacy” refers to an effect determined under highly controlled conditions, while “effectiveness” refers to the effect in a real-world clinical setting^63^. However, focusing exclusively on the conditions under which a trial was conducted without considering the handling of ICE leaves ambiguity^41,60^. Consider a trial where clients receive treatment-as-usual (TAU) or access an IBI in addition to TAU. With TAU, we refer to standard, evidence-based care. The trial recruits patients from general practitioners and uses “a diagnosis of depression” as the sole inclusion criterion. The trial is conducted under uncontrolled conditions, suggesting that it is an “effectiveness trial.” However, the endpoints of patients who stopped using the IBI are modeled as if the discontinuation had not occurred. Is it still an effectiveness trial, although the analysis mimics a scenario of fully adherent clients? To avoid ambiguity, the definitions of efficacy and effectiveness trials should focus on the handling of ICEs^1,41,60^.

### Treatment policy strategy

**Fig. 1:**
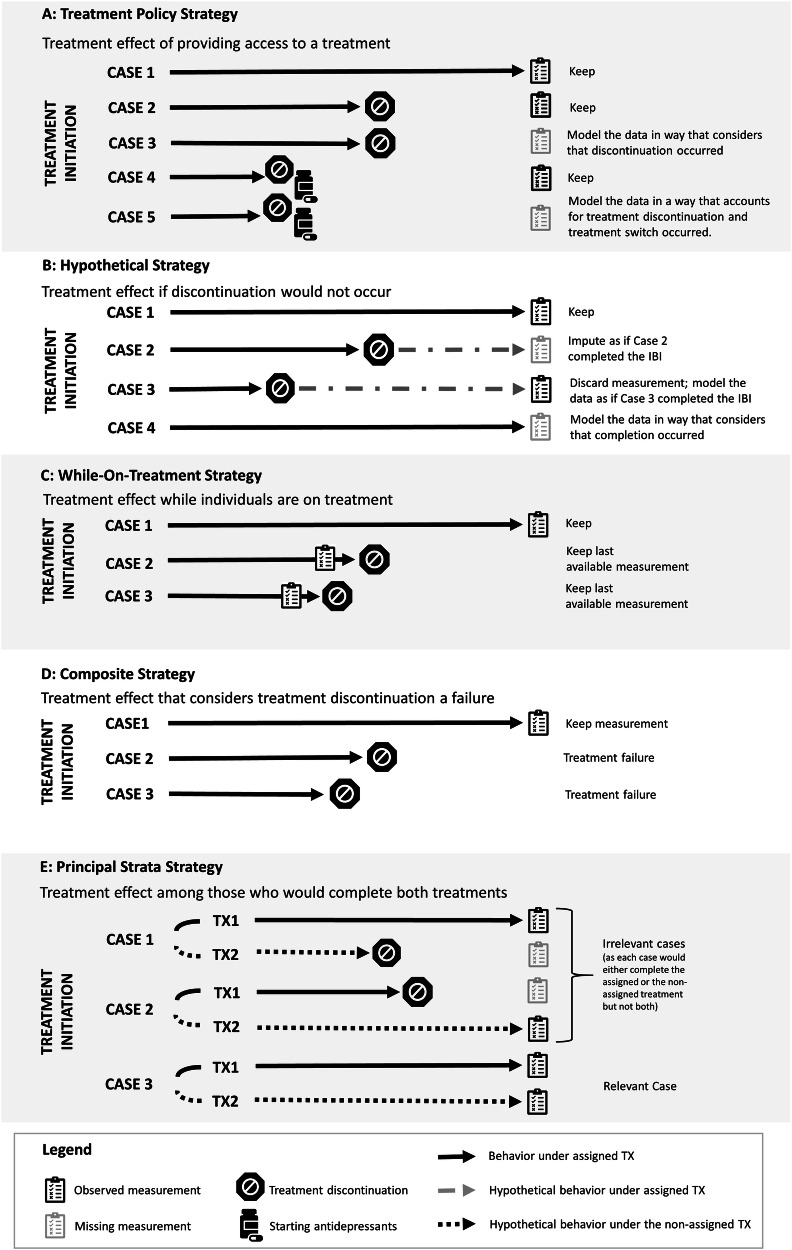
The different strategies applied to treatment discontinuation. The black solid lines indicate behavior until the ICE occurs or until the assessment of the endpoint. **A** Treatment policy strategy: Case 1 (C1) completed treatment and the assessment of the endpoint. C2 and C4 discontinued treatment but completed the endpoint assessment; their measurements are retained because discontinuation is considered part of the treatment regimen. C3 and C5 did not complete the endpoint assessment; therefore, endpoints must be modeled. The model must account for the fact that C3 and C5 discontinued the treatment and that C5 started antidepressants after discontinuation. **B** Hypothetical strategy: C1 completed the treatment and the endpoint assessment. C2 and C3 discontinued the treatment, with C3 completing the endpoint assessment. Given that the trial’s interest in a scenario where discontinuation did not occur, this assessment is non-informative and must be discarded. For both cases, the missing outcomes are modeled to mimic a scenario in which they completed treatment (indicated by gray dashed line). C4 completed treatment but not the assessment. This is “pure” missing data, as it is unrelated to an intercurrent-event (ICE), but still requires modeling. **C** While-on-treatment strategy: C1 completed treatment; therefore, the endpoint assessment is used. C2 and C3 discontinued treatment; thus, only measurements collected before discontinuation are used for analysis. **D** Composite strategy: treatment failure is defined as (**A**) treatment discontinuation or (**B**) less than 50% symptom improvement (composite measure). C1 completed treatment and endpoint assessments; coded as treatment failure if symptom improvement is under 50%. C2 and C3 discontinued treatment. Hence, they are coded as treatment failures. **E** Principal stratum strategy: this analysis focuses on the principal stratum of individuals who *would* complete both treatments, regardless of assignment (TX1: an IBI focusing on cognitive restructuring; TX2: an IBI focusing on behavioral activation). Solid black lines represent behavior under the assigned treatment; dotted black lines represent non-observable behavior under the non-assigned treatment. C1 completed TX1, but *would* have discontinued TX2. C2 discontinued TX1, but *would* have completed TX2. In both C1 and C2, the ICE depends on the assigned treatment. C3 *would* complete both treatments and is thus part of the target stratum. Since statum membership is not observable, it must be estimated.

The treatment policy strategy considers the ICE part of the treatment^1,3^. Measurements remain informative even after an ICE has occurred, making it essential to collect post-ICE measurements^1,3^. Figure 1A illustrates the treatment policy strategy.

By including all endpoints, even those affected by an ICE, in the analysis, the interpretation shifts toward the effect of *providing access to the IBI*, rather than the IBI itself. It quantifies the treatment effect under imperfect adherence.

The EMA recommends this strategy for handling treatment discontinuation in pharmacological trials^21^. This likely extends to IBI trials. Typically, IBI participants decide for themselves how and with what dose they use the treatment material. Therefore, collecting all endpoints at a pre-defined time point, irrespective of the level of engagement, seems to be the natural approach to consider the self-selected heterogeneity in dosage. This strategy bears challenges. Consider a trial that employs this strategy for parallel treatments. Group differences in the endpoint could emerge (or vanish) because the groups initiate parallel treatments with different likelihoods. However, one must assume that the estimate remains informative. High rates of parallel treatments can mask the ineffectiveness of the main treatment component.

The treatment policy strategy aims to minimize the need for assumptions by collecting data from all participants. Participants in IBI trials typically do not attend study centers. This increases the risk of missing endpoints after treatment discontinuation, especially if completing an assessment is tied to the use of the IBI. Without personal contact, individuals may feel less obligated to complete the online assessments. If measurements affected by an ICE are missing, the missing endpoints must be modeled in a way that accounts for the fact that the ICE has occurred^25,26^. Without any information about the post-ICE symptom course, researchers must rely solely on assumptions, such as that the post-discontinuation symptom course parallels that of patients in the control group^17,23,24,27,28^. These assumptions can typically not be verified. Researchers should therefore collect data that is as complete as possible. Thus, strategies to reduce missing values are needed^29,30^. Resources on the treatment policy strategy are available^3,18,23,24,27,31,32^.

### Hypothetical strategy

The hypothetical strategy estimates the treatment effect for a well-defined *what-if* scenario, assuming that the ICE did (or did not) occur^1,3^. Thus, it is impossible to derive a meaningful estimate using observed measurements affected by an ICE. Instead, the endpoints affected by an ICE are modeled in a manner that attempts to mimic the endpoints that would have occurred in the hypothetical scenario^1,3,33^.

Figure 1B illustrates this strategy. The hypothetical strategy shifts the interpretation of the estimate towards an effect that *would have been observed if discontinuation had not occurred*. The strategy relies on two key assumptions: *First*, the hypothetical scenario is clinically meaningful^1,3^. *Second*, given all available data, the symptom course in the hypothetical scenario can be predicted^1,3^. Both are often questionable, especially when the rates with which ICEs occur are large (e.g., discontinuation in IBI trials); therefore, caution is needed. If used, the underlying assumptions must be reported^1,21^.

The following example illustrates possible challenges. A study aims to estimate the effect of a scenario in which all participants complete the IBI (i.e., work with all modules in the intended timeframe). To achieve this goal, one must assume that data from individuals who completed IBI is sufficient to estimate the symptom trajectories of those who discontinued treatment as if they had continued treatment (i.e., the *missing-at-random assumption*, MAR). However, individuals may discontinue the IBI due to dissatisfaction or deteriorating symptoms, and may differ in other characteristics from completers. Therefore, the available information might be insufficient (i.e., we did not collect all relevant covariates) to recover symptom trajectories with continued treatment. The MAR estimates will be biased and may not generalize to real-world settings, particularly when attrition rates are high.

The hypothetical strategy typically relies on some variant of multiple imputations; however, other approaches exist. Several resources are available^3,7,18,31,33^.

### While-on-treatment strategy

The *while-on-treatment* strategy assumes that measurements collected before the ICE occurred contain all relevant information. Measurements after the ICE are irrelevant^1,3^.

Figure 1C illustrates this strategy. The strategy yields an estimate that reflects the treatment effects up to the point at which an ICE occurs.

It is typically less relevant for evaluating the effects of IBI treatment. However, it can be used to assess safety-related aspects, such as the rate of suicides, while following the assigned treatment. However, some aspects warrant attention. First, the strategy may provide an incomplete picture of all adverse events that occur in the context of the treatment^3,34^. In particular, the strategy fails to detect adverse events occurring immediately after discontinuation, such as hospitalization or suicide, which could be a consequence of an ineffective treatment. Second, if people in one treatment arm tend to discontinue the treatment earlier, and the risk of adverse events increases over time, event rates may appear lower due to the lower time on treatment. The interpretability of the between-group differences in rates of adverse events becomes misleading. Therefore, a safety assessment of an IBI should combine the while-on-treatment strategy with assessments collected after discontinuation^34^. Interested readers will find more information elsewhere^3,18,35^.

### Composite strategy

A composite measure is a single outcome variable that combines two or more endpoints^1,3^. The composite strategy considers the ICE part of the endpoint.

Figure 1D illustrates the strategy. The composite strategy shifts the interpretation towards an effect that is a mixture of different components. Consequently, the strategy assumes that the composite measure offers a meaningful interpretation. In particular, it assumes that the components have similar clinical relevance (a limitation that applies to all composite endpoints)^3,36^.

The following example illustrates the interpretative challenges that can emerge when this assumption is violated. Consider two trials that classify a treatment as a failure if (a) there was no reduction in suicidal ideation or (b) hospitalization was required due to suicidal ideation (e.g., rate of treatment failures is the endpoint). Both studies report treatment failure rates of 15%. In study A, 95% of failures were due to no reduction in suicidal ideation, and 5% were due to hospitalization. Study B reports reversed rates. Claiming that both IBIs are equally effective is misleading. Thus, it is essential to report the rate of individual components or to apply appropriate weighting^36,37^. Further problems arise when the likelihood of certain components differs across treatment arms. Consider a trial that compares antidepressants against an IBI. In the antidepressant arm, treatment discontinuation may result from physiological adverse events that can’t occur in IBIs. Thus, the reasons why the ICE occurred must be considered. Composite strategies may fall short of meeting regulatory expectations. Therefore, even when employing a composite strategy, collecting post-ICE data is advisable. More information is provided elsewhere^3,18,36,37^.

### Principal stratum strategy

The principal stratum strategy aims to quantify the treatment effect within the latent stratum of individuals who would (or would not) experience an ICE, regardless of the assigned treatment^1,3^. For instance, in a two-arm trial, one could be interested in the effect among individuals who *would* complete both treatments, irrespective of which treatment arm they are assigned (see ref. ^38^ for further examples)^38,39^. Alternatively, one may focus on individuals who would complete the IBI if assigned to the IBI. Thus, the stratum is defined based on how individuals *would* behave under different treatments^38,39^.

Figure 1E illustrates the strategy. This interpretation of the treatment effects shifts towards the latent population of individuals defined by the (non-)occurrence of the ICE. The estimate no longer quantifies the effect among all randomized individuals^1,5^.

In theory, this strategy can address clinically relevant questions, such as which treatment yields longer-lasting effects among individuals who *would* complete both treatments. Beyond the fact that it can be challenging to define what completion means in IBI trials (see Table 1), it is impossible to observe if an individual will experience the ICE under both treatments. Untestable assumptions are necessary when determining whether an individual belongs to the stratum of interest^3,38,39^. One may attempt to model stratum membership using baseline variables^39^. However, it is impossible to identify stratum membership without error; the statistical analysis must take this into account^3,38,39^.

Kahan et al. discuss rare instances in which strata appear naturally^40^. Consider a study comparing guided versus unguided IBI. Some individuals discontinue the treatment before learning their assigned trial arm. Since discontinuation is unrelated to the treatment, excluding them from the analysis won’t introduce bias^40^. The interpretation shifts toward the subset of individuals *who would always start the assigned treatment* (=the principal stratum)^40^. Guidance on appropriate statistical approaches is provided elsewhere^3,7,18,38,39^.

## Two examples

Several applications of the framework to pharmacological treatments have been published^11–14,41–44^. Supplementary Tables 1 and 2 present two fictitious examples of IBI trials. They are not intended as best-practice examples, but rather to illustrate the principles of the addendum. The example follows the template to describe trials provided by Ratitch et al.^8,9^.

## Discussion

This manuscript introduced the ICH E9(R1) addendum to IBI researchers. The addendum prompts prioritizing the collection of measurements vital for answering the clinical question and anticipating how ICEs impair the availability of needed measurements^1^. When ICEs are anticipated, trial design and statistical approach can be aligned to derive informative estimates. Trial protocols should report the targeted estimand^1,22,45^. Reports of completed trials should provide sufficient detail to derive the estimand^20,46^. This includes describing estimated effects with precise language. Instead of claiming that the “IBI was effective,” one might report that “group differences reflect the effect that *could* be achieved if all individuals *would be* compliant” when the hypothetical strategy was employed^18^.

Standardized reporting of estimands will facilitate the synthesis of findings in meta-analyses^6^. Currently, the lack of clarity in reporting estimands hampers systematic evaluations of how estimands explain between-study heterogeneity^20,46^. To increase understanding of estimands, helpful teaching materials have been published^16,26,47^.

While this manuscript focuses on parallel group trials, the framework extends to factorial designs^48^, cluster randomized trials^49^, and non-inferiority and equivalence trials^42,50–52^. It should also be considered in face-to-face psychotherapy trials.

## Supplementary information

Supplementary information

## Data Availability

No datasets were generated or analyzed during the current study.
