# Supplementary material for: Using the ICH estimand framework to improve the interpretation of treatment effects in internet interventions
Source: NPJ Digit Med. 2025 Aug 20;8:535. doi: 10.1038/s41746-025-01936-0 (PMC12368201; doi:10.1038/s41746-025-01936-0)
Supplement: Supplementary file 1 — Supplementary information [file 41746_2025_1936_MOESM1_ESM.pdf]

**Supplemental Table 1.** Detailed description of the trial design for Example 1.
**Overall Description**
A health insurance company wants to evaluate the benefits of providing individuals seeking psychotherapy for a first episode of major depression access to an IBI. While previous studies have shown that the IBI under investigation is superior to a waiting list control, the aim is now to investigate whether offering IBI could be a valid interim solution associated with a reduction in depressive symptoms three months after randomization.<br><br>To address this research question, the RCT allocates participants in a 1:1 ratio to one of two treatment arms. Eligible participants are adults who (a) have an indication for psychotherapy based on a diagnosis of depression, (b) refuse to receive antidepressants at the time-point of diagnosis, (c) are not currently receiving depression-related treatment, and (d) have started to seek psychotherapy (<u>*population*</u>).<br><br>To ensure that eligibility and randomization remain aligned, the health insurance company collaborates with general practitioners who offer individuals participation if a mild to moderate depression is diagnosed. Participants in the experimental arm receive immediate access to a self-guided IBI, which offers a flexible, non-sequential selection of CBT-based treatment modules. Participants in the control arm receive access to the IBI after a 3-month waiting period. All participants will be encouraged to seek psychotherapeutic support and/or initiate any depression-related treatments they deem appropriate. Therefore, all depression-related treatments initiated after randomization will be considered as part of the treatment regimen under investigation, even if individuals choose a treatment other than psychotherapy. This liberal definition of the treatment regimen takes into account that some individuals may start psychotherapy but change their treatment preferences after some time (<u>*treatment*</u>). Depressive symptoms will be assessed using the PHQ-9 three months after randomization (<u>*endpoint*</u>). A statistically lower mean depressive symptom score would be interpreted as evidence of benefit (<u>*population-level summary*</u>).<br><br>All randomized individuals receive financial compensation for completing brief telephone-based assessments every four weeks, which evaluate symptom severity, treatment use, and current efforts to seek support. Additionally, the trial collects information on reasons for discontinuing the search for support, if applicable.
**Identify the decision maker:**
The insurance company.
**Define objective:**
Determine if providing individuals access to an IBI is associated with lower depressive symptom scores three months after randomization in comparison to individuals without access to an IBI.
**Possible ICEs:**
Different ICEs may occur:<br><br>Experimental condition (IBI access + search for psychotherapy):<br><br>1. Discontinuation of the IBI (i.e., stopping to work with the provided IBI)<br>2. Treatment non-adherence (i.e., not using the IBI as intended or irregularly)<br><br>Control condition (search for psychotherapy)<br><br>3. Discontinuing the search for psychotherapy because of knowing that they get access to IBI after three months.<br><br>Both treatment conditions:<br><br>4. Discontinuing the search for psychotherapy due to demotivation, symptom improvement, or any rescue treatment (e.g., hospitalization due to elevated suicidality)<br>5. Discontinuing the search for psychotherapy due to the initiation or expected initiation of a depression-related treatment shortly.<br><br>ICEs (1) and (2) are not problematic, as the stakeholders' primary interest is in quantifying the effect of *providing individuals access* to an IBI. Therefore, measurements among these individuals should be collected regardless of how they adhere to the IBI.<br><br>ICE (3) is problematic because individuals give up seeking psychotherapy because of subsequent access to IBI. Such behavior would not occur in a real-world setting where the IBI under investigation is not available

(i.e., in the control condition of clinical interest). If many individuals in the control group show such behavior, the benefit of providing access to the IBI could be overestimated, as individuals in the control group remain untreated. Consequently, measurements collected from these individuals require special treatment.

ICE (4) is not problematic because discontinuing the search for treatments due to symptom improvement, demotivation, or the need for a rescue treatment reflects natural behavior in a real-world clinical setting.

ICE (5) is unproblematic because the initiation of a depression-related treatment is consistent with the study design, which considers that individuals may change their treatment preference.

To differentiate ICEs (3) to (5) in the data analysis, participants are asked at each assessment whether they are still seeking support and, if not, why they stopped.

Treatment Regimen Under Investigation

The treatment regimen under investigation provides individuals access to an IBI after they have received an indication for psychotherapy. All forms of IBI utilization and all additional or alternative depression-related treatments initiated are considered valid treatment components.

The attributes of the estimand:

Population: Self-selected adults (≥18). Individuals who (a) have an indication for psychotherapy based on a diagnosis of depression, (b) are not currently receiving depression-related treatment, and (c) have started to seek psychotherapy. Comorbidities are allowed. However, individuals experiencing acute suicidal ideation or other conditions (e.g., a current psychotic episode) that require immediate inpatient treatment are excluded.

Endpoint: Self-reported symptoms of depression (PHQ-9) three months after randomization. We consider a time frame of ±7 days to be appropriate for assessing the primary endpoint and all within measurements.

Handling of ICEs:

- ICEs 1, 2, 4, and 5 will be treated with the *treatment policy strategy*, as the trial is interested in the effect of providing individuals access to an IBI. Therefore, the study will consider treatment discontinuation and non-compliance with IBI as part of the treatment regimen in the IBI arm and the use of alternative treatments and discontinuation of support-seeking due to demotivation, symptom improvement, rescue treatment, or the (expected) initiation of any depression-related treatment as part of the treatment regimen in the IBI arm and the control arm.
- Measurements of individuals assigned to the control condition who discontinued their psychotherapy search because of subsequent access to IBI (ICE 3) are excluded from the analysis and modeled using a *hypothetical strategy*. The hypothetical strategy aims at a scenario in which the individuals would not have given up seeking support because they knew they would get access to IBI. The MAR assumption is applied: It is assumed that the data collected from other individuals in the control arm who behaved as intended are sufficient to recover the measure affected by this ICE for the hypothetical scenario in which they continued to seek support and may or may not have found support, or stopped searching for support due to demotivation or improvements.

Summary statistic: The unadjusted mean difference in PHQ-9 scores 3 months after randomization. That is, the estimand considers that the group difference measured at three months after randomization is a blend of various effects. The rate of ICE should be considered when interpreting the finding.

Data that is useful for the primary estimand:

The study tries to follow up with all patients. However, measurements affected by ICE (3) in the control arm are excluded from the analysis and handled using a hypothetical strategy. Whenever individuals affected by ICEs 1, 2, 4, and 5 have missing data, the imputation tries to consider that the ICE has occurred.

Retention strategies:

Retention strategies aim to obtain the most complete data possible.

a. Participants are informed that their data is valuable, no matter how they use the IBI.
b. Participants receive money to complete the assessments.
c. All assessments are conducted via telephone to simulate a scenario similar to a study center.
d. Participants can choose their preferred follow-up appointment via an online scheduler.
e. The study team proactively contacts participants who do not choose an appointment.
f. A very short and focused assessment is used.

**Main estimator:**
Between-group differences in depression severity 3 months after randomization will be tested with a *t*-test for significance. If necessary, the nesting of individuals within general practitioners is considered. Given that some data will be imputed, estimates are pooled across all multiple imputed datasets using Rubin's rules. Results will be reported in units of PHQ-9 points to avoid standardization.
**Missing-data assumptions:**
A complete observation includes measurements at four time points: Week 0, 4, 8, and 12 (primary endpoint).<br><br>At each measurement occasion, the symptom severity and the current treatment status will be assessed. This includes a question about whether individuals still search for support. If they stopped, the reasons why will be investigated. Nevertheless, it is unlikely that all participants will have complete data at all time points.<br><br>*Missing value concerning the current treatment status.* Whenever participants skip measurement occasions, the treatment status in the skipped measurement occasions will be assessed at later measurement occasions. Whenever participants report a treatment at week 4 or 8 but never return for further assessments, it is assumed that the treatment remains the same at a later measurement occasion. If the treatment status remains unclear because participants were unavailable for follow-ups and did not report receiving a treatment in the last available measurement, the missing treatment status is imputed under the MAR assumption.<br><br>*Missing symptom ratings:* All missing symptom ratings (i.e., due to skipped measurement occasions or monotone missing data patterns, including those of the primary endpoint) will be imputed under the MAR assumption. It is assumed that the available baseline measurements, (imputed) intermediate measurements, and (imputed) treatment status are sufficient to recover the symptom ratings properly.
**Sensitivity analysis:**
We perform a delta adjustment/tipping point analysis. We progressively worsen the imputed endpoints of the IBI arm until the conclusion regarding the effectiveness change. This analysis stresses the assumption that imputations under MAR in the IBI condition are too optimistic (i.e., the data is not missing at random)
**Sample size:**
Although prior evidence suggested a moderate effect size (*d* = 0.50) compared to a waitlist control, the trial anticipates a smaller between-group effect (*d* = 0.30) due to potential concurrent initiation of psychotherapy or other depression-related treatments in both treatment arms. When calculating the sample size, the proportion of data that must be imputed under the MAR assumption (measurements affected by ICE 3, missed endpoint assessments) must also be taken into account. Both aspects will inflate the needed sample size; see Fang and Jin[1] for suggestions on incorporating ICEs into the computation of the sample size.

Supplemental Table 2. Detailed description of the trial design for Example 2.

General Description

The RCT aims to inform evidence-based treatment recommendations for individuals experiencing a first episode of mild major depression. The trial seeks to answer whether offering antidepressants *or* providing access to an IBI leads to larger symptom reduction within eight weeks after randomization.

Participants are allocated to two treatment arms in a 1:1 ratio. Eligible participants are adults diagnosed with a first episode of mild major depression who consider both antidepressants and IBI viable treatment options (*population*). In the antidepressant arm, participants receive monotherapy with antidepressants prescribed by a study clinician. In the IBI arm, participants will receive access to a CBT-based IBI for depression guided by study clinicians. Substantial efforts will be made to keep participants as adherent as possible (treatment). The endpoint is depression severity (PHQ-9) eight weeks after randomization (*endpoint*). A baseline-adjusted between-group difference of about one point on the PHQ-9 scale is considered clinically meaningful (*population-level summary*).

During the 8-week intervention period, telephone check-ins and assessments will be conducted in weeks 2 and 6. In week 4, participants will have an in-person meeting with the clinician. In each assessment, the (a) current depressive symptoms using PHQ-9, (b) self-reported adherence to the assigned treatment, and (c) the use of concurrent treatments will be assessed. Although the trial is mainly interested in comparing IBI against antidepressants, it also aims to follow up all participants, including those who discontinued or changed the assigned treatment, allowing for a secondary estimand. To ensure complete data, participants receive financial compensation for completing assessments, even if they discontinue the assigned treatment prematurely.

Identify the decision maker:

Institutions that develop treatment guidelines for depression.

Define objective:

Decide if adults with a first depressive episode of mild depression should be offered antidepressants or access to a guided IBI.

Possible ICEs:

The same ICEs are relevant for both treatment arms.

1. Treatment discontinuation with or without starting a new treatment after discontinuation without clinical necessity
2. Treatment switching with clinical necessity (e.g., initiating rescue treatment)
3. Starting a treatment in parallel

ICE (1) refers to treatment discontinuation and a possible change in treatment (i.e., participants start a new treatment outside the trial) without clinical indication. In the antidepressant arm, discontinuation occurs when people report that they are no longer taking antidepressants; in the IBI arm, discontinuation occurs when they report that they have decided to stop using the IBI. This ICE also considers individuals who discontinue treatment (and change it) due to mild side effects (e.g., dry mouth), a lack of benefit, or sufficient improvement. This ICE is problematic as these individuals may have benefited from continuing the assigned treatment. We anticipate that the rate of individuals who do so is low (~5%) due to the short observation period, the population that considers both treatments feasible, and regular appointments with the clinician.

ICE (2) is, in principle, not problematic because initiating a rescue treatment is clinically indicated. However, in the present trial, it is problematic in the sense that we are interested in comparing IBI against antidepressants, that is, the treatment effect that could be observed if rescue treatment was not made available. Therefore, a hypothetical strategy for handling the ICE is needed. However, given the focus on individuals with mild depression, we expect that the rate of individuals experiencing this ICE is low.

ICE (3) is problematic as the trial is specifically interested in comparing IBI against antidepressants. Starting a treatment in parallel to the assigned treatment will blur the interpretation of the between-group difference. Therefore, a specific strategy to handle the ICE will be needed. Again, we expect the rate of individuals experiencing this ICE to be low due to the short observation period.

Imperfect adherence is not considered problematic. This includes patients who take pills or log in to the IBI platform irregularly. However, we encourage patients to use the treatment as prescribed. To increase adherence, individuals in the IBI arm receive encouraging reminders to log in. Moreover, the IBI enables patients to select the most appealing treatment modules, thereby maintaining high engagement levels. Furthermore, the

IBI is guided, which typically leads to higher adherence. Individuals in the antidepressant arm receive a supporting app helping them to adhere to the medication schedule.

Treatment Regimen Under Investigation

The treatment regimens under investigation are treatments in which only antidepressants (Arm 1) or the IBI (Arm 2) are available for treating symptoms of depression, and in which individuals do not discontinue the randomized treatment for eight weeks. However, they may use the assigned treatment imperfectly. We focus on treatment regimens in which rescue treatment is not made available, even if clinically indicated (note, however, that in the trial, rescue treatment will of course be made available).

The attributes of the estimand:

Population: Adults (≥ 18 years) diagnosed with a first episode of mild depression according to DSM-5 criteria, who are not currently receiving any treatment for depression, report no symptoms of acute suicidality, and consider both IBIs and antidepressants as viable treatment options.

Endpoint: Differences in depression severity, as measured by the PHQ-9, eight weeks after initiation of the randomized treatment. A time window of ±7 days around the 8-week mark is considered acceptable.

Treatment of intercurrent events:

- ICE1: These patients could have continued the assigned treatment, but either discontinued it without further treatment or actively switched to another treatment without a clinical indication. To address these ICEs, a *hypothetical strategy* using multiple imputations will be employed. Multiple imputations are favored over other approaches due to their flexibility in incorporating auxiliary information. The MAR assumption will be imposed: The imputation model will consider all information collected from all individuals while they are on treatment (e.g., symptom measurements and usage behavior) to recover the symptom course for the hypothetical scenario of not discontinuing the treatment.
- ICE 2: The last-observation-carried-forward (LOCF) strategy is applied for individuals who discontinued or changed the assigned treatment due to a clinical indication (e.g., those who experienced serious adverse events). In particular, the last symptom assessment collected immediately before the need for rescue treatment will be carried forward. We assume that these individuals would not have experienced further improvement had they continued the assigned treatment (*hypothetical strategy*). Note, however, that a second estimand that treats this differently could be added (see end of this table)
- ICE 3: All measurements collected after initiating an add-on treatment are discarded and treated as if using a *hypothetical strategy*. The MAR assumption is imposed. We assume that the information collected from individuals who did not start an add-on treatment is sufficient to recover the symptom developments under the hypothetical scenario. Note that this could lead to estimates that are clinically not informative if the rate and the characteristics of individuals who initiate an add-on treatment differ between treatment arms. These differences in rates may mask an ineffective main treatment ingredient. Therefore, information on why individuals started the add-on treatment (i.e., due to adverse events or lack of efficacy) should be considered when interpreting the findings.

Summary statistic: The baseline-adjusted mean difference in PHQ-9 scores eight weeks after randomization.

Data that is useful for the primary estimand:

Only data from individuals who (a) use the randomized treatments (imperfectly), (b) did not change the treatment without clinical indication, (c) did not start a parallel treatment, and (d) report their outcome in the specified time frame are useful for the primary estimand. However, we plan to examine a secondary estimand that handles all ICE with a treatment policy strategy. Therefore, we will follow up with all individuals in week 8.

Retention strategies:

Several retention strategies are employed

1. The consent form explains the need to collect data.
2. Participants get a financial incentive to complete assessments.
3. The trial uses a focused and short assessment.
4. Measurements are collected via telephone (weeks 2, 6, 8) or by a clinician in an in-person meeting (week 4).
5. Moreover, to increase adherence to the assigned treatment, individuals are informed that they will receive support to change the treatment or use a non-assigned treatment as an add-on or stand-alone treatment immediately after the 8-week study period.

Main estimator:

Between-group differences in depression severity eight weeks after randomization are estimated using ANCOVA-like regression with a dummy-coded treatment condition adjusted for baseline depression symptom severity. The model will be estimated in each imputed dataset and pooled using Rubin's rules. Group differences will be reported in units of PHQ-9 points (unstandardized).

Missing data assumptions:

Data of intermittent missing data points (e.g., of adherent individuals who skipped any of the measurement occasions), as well as for individuals who did not provide the primary endpoint, are imputed under the MAR assumption. It is assumed that the information collected while individuals use the assigned treatment is sufficient to accurately recover the symptom scores.

Sensitivity analysis:

The imputation model assumes that the available information is sufficient to recover the endpoints for individuals whose measurements are (a) affected by ICEs, (b) handled using a hypothetical strategy, and (c) for those with missing data. However, this assumption could be overoptimistic and may lead to overestimating symptom improvements within each arm. The amount of bias may vary if attrition rates differ between treatment arms. Therefore, a proper sensitivity analysis is needed. To examine the robustness of our conclusions to violations of the MAR assumption, we will apply a tipping point analysis (delta adjustment). Specifically, we will progressively worsen the values imputed under MAR by 2.5%, 5%, 7.5%, and 10% until the conclusions change.

Sample size:

We calculate power for a two-sided t-test. We conservatively assume that including the baseline symptom severity as a covariate in the ANCOVA model does not increase precision. Considering that most ICEs are handled with a hypothetical strategy, the resulting sample size is increased by $1/(1 - r_{MIS})$, r_{MIS} = rate of individuals with missing data or ICEs handled with the hypothetical strategy[1].

Additional considerations:

- We construct a secondary estimand that treats all ICEs with a treatment policy strategy. To do so, we attempt to follow up with all individuals 8 weeks after randomization, including those who required hospitalization or discontinued the treatment. This approach considers treatment discontinuation, parallel treatments, and the initiation of a rescue treatment as part of a real-world strategy (i.e., continue the assigned treatment unless a clinical indication to do so arises). This estimand will provide an interesting supplemental perspective.
- A supplemental analysis focuses on the stratum of individuals who would complete the treatment regardless of which treatment arm they are assigned to (principal stratum).

Reference:

1. Fang, Y. & Jin, M. Sample Size Calculation When Planning Clinical Trials with Intercurrent Events. *Ther. Innov. Regul. Sci.* **55**, 779–785 (2021).
